# Association between Circulating T Cells and the Gut Microbiome in Healthy Individuals: Findings from a Pilot Study

**DOI:** 10.3390/ijms25136831

**Published:** 2024-06-21

**Authors:** Sithara Vivek, You Shan Shen, Weihua Guan, Guillaume Onyeaghala, Mosunmoluwa Oyenuga, Christopher Staley, Amy B. Karger, Anna E. Prizment, Bharat Thyagarajan

**Affiliations:** 1 Department of Laboratory Medicine and Pathology, University of Minnesota, MMC 609, 420 Delaware Street, Minneapolis, MN 55455, USA; svivek@umn.edu (S.V.);; 2 Division of Biostatistics, School of Public Health, University of Minnesota, Minneapolis, MN 55455, USA; 3 Hennepin Healthcare Research Institute (HHRI), Minneapolis, MN 55404, USA; 4 Department of Internal Medicine, Abbott Northwestern Hospital, Minneapolis, MN 55407, USA; 5 Department of Surgery, University of Minnesota, Minneapolis, MN 55455, USA

**Keywords:** microbiome, T cells, healthy gut

## Abstract

Though the microbiome’s impact on immune system homeostasis is well documented, the effect of circulating T cells on the gut microbiome remains unexamined. We analyzed data from 50 healthy volunteers in a pilot trial of aspirin, using immunophenotyping and 16S rRNA sequencing to evaluate the effect of baseline T cells on microbiome changes over 6 weeks. We employed an unsupervised sparse canonical correlation analysis (sCCA) and used multivariable linear regression models to evaluate the association between selected T cell subsets and selected bacterial genera after adjusting for covariates. In the cross-sectional analysis, percentages of naïve CD4+ T cells were positively associated with a relative abundance of *Intestinimonas*, and the percentage of activated CD8+ T cells was inversely associated with *Cellulosibacter*. In the longitudinal analysis, the baseline percentages of naïve CD4+ T cells and activated CD4+ T cells were inversely associated with a 6-week change in the relative abundance of *Clostridium_XlVb* and *Anaerovorax*, respectively. The baseline percentage of terminal effector CD4+ T cells was positively associated with the change in *Flavonifractor*. Notably, the microbiome taxa associated with T cell subsets exclusively belonged to the *Bacillota phylum*. These findings can guide future experimental studies focusing on the role of T cells in impacting gut microbiome homeostasis.

## 1. Introduction

The gut microbiome, which is the collection of a diverse community of microorganisms, including bacteria, viruses, and fungi that live in the digestive tract, plays an important role in the development and function of the immune system. The gut microbiome can influence the differentiation and activation of immune cells, such as T cells and B cells [1,2,3], and also the production of immune molecules, such as antibodies and cytokines [4,5]. Different bacterial genera may have different functions and effects on the immune system, depending on the context and other factors such as diet or disease conditions. *Bacillota* and *Bacteroidota* are the two major phyla found in the gut. Certain bacterial genera within the *Bacillota* phylum affect T cell immunity in the local environment. For instance, *Lactobacillus* promotes the production of Th1 cells that play a key role in the host defense against intracellular pathogens [6]. Additionally, *Bifidobacterium*, which belongs to the *Actinomyceota* phylum, downregulates the production of Th2 and Th17 cells, upregulates the production of interferon beta, and increases the number and suppressive function of regulatory T cells (Tregs) [7,8,9,10]. In contrast, *Bacteroides*, one of the most abundant genera under phylum *Bacteroidota*, is found to promote Th17 cell differentiation and excessive Th17 cell activity, which play a critical role in the host defense against extracellular bacteria and fungi [11,12]. Thus, a healthy gut microbiome can help to promote a well-functioning immune system by strengthening the barrier function of the gut [13], producing anti-inflammatory molecules, and training the immune system to recognize and respond to pathogens.

Though the critical role of the microbiome in shaping the immune system is well recognized, there is now substantial evidence that there is a bidirectional relationship between the microbiome and both the innate and adaptive immune systems [14]. Prior studies have documented the role of tissue resident innate and adaptive immune cells in the intestinal mucosa in shaping the intestinal microbiome composition [15,16]. Specifically, the intestinal innate immune system secretes antimicrobial peptides and pathogen recognition receptors (e.g., Toll-like receptors) that play important roles in shaping the intestinal microbiome [17]. In addition, the intestinal adaptive immune system consisting of both B cells and T cells shapes the intestinal microbiome by producing IgA-specific responses against commensal bacteria via both T cell-dependent and T cell-independent mechanisms [18]. While the role of the local intestinal immune system in shaping the gut microbiome has been studied in previous studies, the role of circulating T cell subsets in peripheral blood in shaping the gut microbiome has not been previously evaluated.

In this study, we collected blood and repeated stools samples in a clinical trial of healthy participants and measured immune cells at baseline to comprehensively characterize the associations between the percentages of peripheral blood T cell subsets and the relative abundance of gut microbiomes. We hypothesized that a higher proportion of T cell subsets such as naïve CD4+ T cells that have been previously associated with lower multimorbidity and mortality will be associated with bacterial taxa that reduce inflammation and are beneficial for human health, while a higher proportion of circulating T cells subsets such as effector and activated T cells that indicate a recent or ongoing immune response will be associated with bacterial taxa that are detrimental to human health.

## 2. Results

The study participants had a mean age of 61.3 ± 5.1 years and mean BMI of 27.7 ± 4.5 kg/m^2^. Females represented 64% (N = 32) of the participants, and 60% (N = 30) of the participants received aspirin during the clinical trial. Aspirin intake was not associated with any of the T cell subsets studied. The sCCA was performed separately for the cross-sectional and longitudinal (6-week microbiome change) analyses between 10 T cell subsets and 50 gut bacterial genera and computed 10 canonical variates. The canonical correlation coefficients between different T cell subsets and bacterial genera ranged from 0.47 to 0.69 in the cross-sectional analysis (Supplementary Table S1) and from 0.26 to 0.73 in the longitudinal analysis (Supplementary Table S2). In the cross-sectional analysis, we found three significant canonical variates (Pearson correlation coefficient: K1 [r = 0.69, *p* = 0.007]; K3 [r = 0.64, *p* = 0.003]; and K5 [r = 0.60, *p* = 0.045] Table S1) and one in the 6-week change analysis (K1 [r = 0.73, *p* = 0.006] Table S2).

### 2.1. Cross-Sectional Association between the Percentage of T Cell Subsets and Relative Abundance of Bacterial Genera

**Figure 1:**
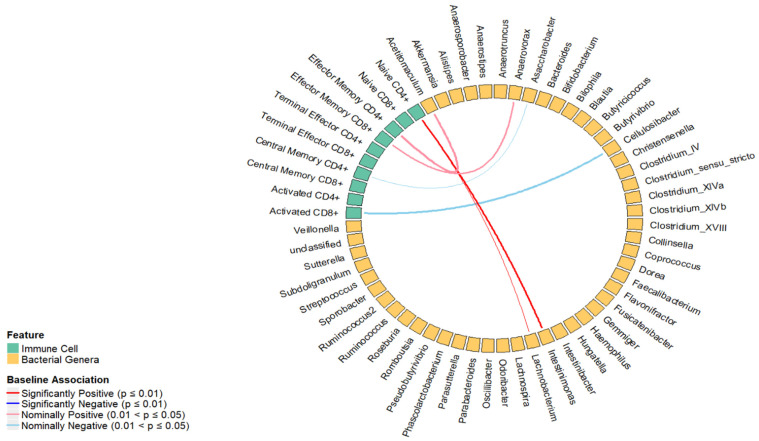
Results of the cross-sectional association between T cell subsets and bacterial genera from significant canonical variates after adjusting for covariates. The blue line indicates an inverse association, and the red line indicates a positive association between a T cell subset with bacterial genera.

In the cross-sectional analysis, canonical correlation analysis between T cell subsets and bacterial genera from three significant canonical variates identified 98 pairs of bacterial genera and T cell subsets for further linear regression analysis (Supplementary Table S1). As shown in Figure 1, we found that the percentage of naive CD4+ T cells was positively associated with *Intestinimonas* (β = 0.03, *p* = 0.01). Though not statistically significant at the pre-determined significance level of 0.01, we found a nominal association between the percentage of naive CD4+ T cells and *Lachnobacterium* (β = 0.02, *p* = 0.01), central memory CD4+ T cells and *Asaccharobacter* (β = −0.02, *p* = 0.05), effector memory CD4+ T cells and *Acetitomaculum* (β = 0.05, *p* = 0.04), effector memory CD8+ T cells and *Anaerovorax* (β = 0.03, *p* = 0.05), and activated CD8+ T cells and *Cellulosibacter* (β = −0.11, *p* = 0.02). The univariate and multivariable regression results are shown in Figure 1 and Table 1, respectively.

### 2.2. Longitudinal Analysis Percentage of T Cell Subsets at the Baseline and Fold Change of Bacterial Genera over 6 Weeks

**Figure 2:**
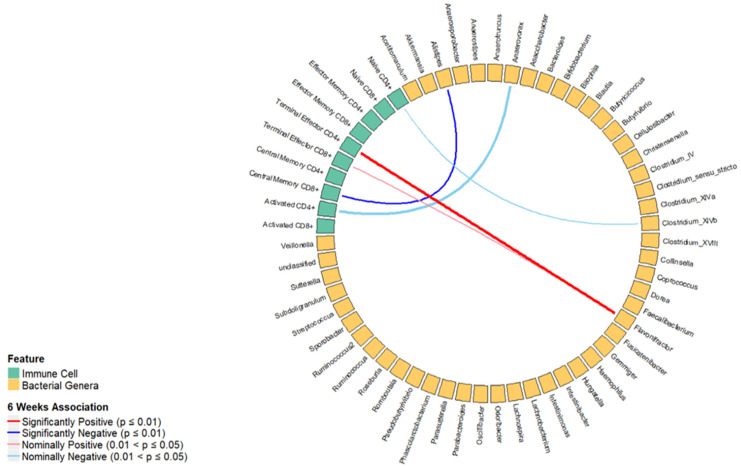
Results of the longitudinal association between T cell subsets and bacterial genera from significant canonical variates after adjusting for covariates. The blue line indicates an inverse association, and the red line indicates a positive association between a T cell subset with bacterial genera.

In the longitudinal analysis, 108 pairs of bacterial genera and T cell subsets were identified from significant canonical variates (Supplementary Table S2). We found that terminal effector CD4+ T cells were positively associated with *Flavonifractor* (β = 0.06, *p* < 0.01). Though not statistically significant at the 0.01 significance level, the terminal effector CD8+ T cells were nominally associated with *Flavonifractor* (β = 0.02, *p* = 0.03). We found inverse associations between the baseline percentages of central memory CD8+ and *Alistipes* (β = −0.05, *p* = 0.01) and naive CD4+ T cells and *Clostridium XlVb* (β = −0.02, *p* = 0.03) and activated CD4+ T cells and *Anaerovorax* (β = −0.06, *p* = 0.04). The results of the univariate regression analysis are shown in Figure 2, and the multiple linear regression analysis is shown in Table 2.

In both cross-sectional and longitudinal analyses, we found that the percentages of naive CD4+ T cells were positively associated with the relative abundance of *Intestinimonas* and nominally associated with *Clostridium XlVb* (Table 3). Of note, the majority of bacterial genera that displayed significant associations with T cell subsets were predominantly found within the *Bacillota* phylum.

## 3. Discussion

Though the link between the composition of the gut microbiome and immune cells with human health and disease has been previously investigated in multiple studies [14,16,19], the connection between immune subsets in the peripheral blood and gut microbiome in healthy individuals remains largely unexplored. In this study, we showed that baseline percentages of circulating T cell subsets in peripheral blood were associated with changes in the relative abundance of bacterial genera over 6 weeks of follow-up in healthy participants enrolled in a clinical trial. To the best of our knowledge, this is the first study that has evaluated the association between T cell subsets in peripheral blood and change in the relative abundance of gut bacteria in healthy participants. We utilized a sparse canonical correlation analysis (sCCA) to evaluate the relationship between high dimensional gut microbiome data and the comprehensive adaptive immune T cell phenotype by preserving the main facets that explain the correlation between two feature sets. sCCA is commonly used in genomics [20,21] or neuroscience [21,22,23] research areas to analyze high-dimensional data. This method allowed us to evaluate a selected set of T cell subsets and bacterial genera that had the highest correlation in the canonical component.

In our cross-sectional analysis, we found that a higher proportion of naïve CD4+ T cells was associated with an increased relative abundance of bacterial genera *Intestinimonas*. *Intestinimonas* is a genus within the *Oscillospiraceae* family, and previous studies have shown that certain species of *Intestinimonas* may play a role in alleviating inflammatory and metabolic diseases [24,25]. A previous study showed that a higher relative abundance of *Intestinimonas* genera is associated with elevated levels of IL-4, suggesting that alterations in the composition of the gut microbiota may play a role in contributing to systemic inflammation in patients with Huntington’s disease [24]. This indicates *Bacillota*-derived molecular patterns like lipoteichoic acids, lipoproteins, and peptidoglycan are recognized by TLR2 heterodimers (TLR2/TLR1 or TLR2/TLR6), which allow the innate immune system to sense and respond to infections by these Gram-positive bacteria [26]. The TLR2-mediated signaling upon binding these ligands results in the production of inflammatory cytokines and the activation of immune responses against *Bacillota* pathogens [27]. Another study identified that treatment with Chinese caterpillar fungus polysaccharides was associated with an increase in short-chain fatty acid (SCFA)-producing bacteria within the gut microbiome, including *Intestinimonas butyriciproducens*, leading to a higher production of SCFAs, which, in turn, played a role in reducing elevated glucose levels [25]. Thus, a higher relative abundance of *Intestinimonas* appears to be beneficial for human health. We have previously shown that lower naïve CD4+ T cells are a biomarker for an age-related immune phenotype (ARIP) and are associated with an increased risk of age-related morbidity and mortality [28]. Hence, the positive correlation between *Intestinimonas* and the percentage of naive CD4+ T cells in the cross-sectional analysis suggests that ARIP markers and intestinal microbial abundance may contribute to future human health. In addition, we found that the proportion of activated CD8+ T cell subsets was inversely associated with the relative abundance of *Cellulosibacter*. The dysregulation or excessive activation of CD8+ T cells can lead to various immune-mediated diseases, including autoimmune disorders and chronic inflammatory conditions. For instance, the dysregulation of CD8+ T cell activation can contribute to the development of autoimmune diseases such as autoinflammatory diseases and chronic autoimmune conditions, leading to increased morbidity and mortality [29,30]. The balance of T cell activation is crucial, as it can result in either tolerogenic or proinflammatory outcomes, impacting the immune response and the development of immune-mediated diseases.

In our longitudinal analysis, we observed that a reduced proportion of activated CD4+ T cells were inversely associated with the relative abundance of *Anaerovorax*. Limited research has investigated the impact of *Anaerovorax* on host health status [31,32,33]. A study on children showed that moderate-to-vigorous physical activity was associated with a higher relative abundance of *Anaerovorax* [32], indicating that a higher relative abundance of *Anaerovorax* may confer potential health benefits. Conversely, elevated levels of activated CD4+ T cells, which indicate a negative prognosis, were observed during the acute phase of HIV-1 infection [34]. In general, it has been hypothesized that the higher frequencies of activated T cells of various phenotypes could serve as biomarkers of poorer outcomes in several conditions, including vaccine-induced protection (at defined time point(s) post-vaccination), disease activity (for T cell-mediated autoimmune or infectious diseases), and chronic inflammation (as has been shown in HIV infection) [35,36]. Consequently, the observed negative association between activated CD4+ T cells and *Anaerovorax* in our longitudinal analysis is consistent with the health-promoting behaviors associated with *Anerovorax* and the poor health outcomes associated with activated CD4+ cells. Hence, this finding warrants further investigation into their associations with health and disease states. The longitudinal analysis also showed that reduced levels of CD4+ naive T cells are associated with increased levels of *Clostridium XIVb*. *Clostridium XlVb* has demonstrated both positive and detrimental effects on human health and disease conditions. In two studies on ulcerative colitis, *Clostridium XlVb* was beneficial to the immune response or could reduce the risk of developing *Clostridioides difficile* infection [37,38]. Conversely, studies on cognitive function revealed harmful effects of *Clostridium XlVb* in individuals with type 2 diabetes mellitus and Parkinson’s disease [39,40]. While a reduced proportion of naïve CD4+ T cells, an ARIP marker, has been associated with an increased risk of multimorbidity and short-term mortality, the biological significance of the observed association with an increase in *Clostridium XIVb* should be further evaluated. In addition, we observed an elevated proportion of terminal effector CD4+ cells positively associated with *Flavonifractor* in the 6-week follow-up. *Flavonifractor* has proinflammatory properties that were found in higher abundance in patients with various brain-related diseases [41]. Our previous study showed that CD4+ effector memory T cells are associated with lower immune function in aging and are associated with type 2 diabetes, which is itself proinflammatory. Another study found that *Flavonifractor plautii* was found to increase the expression of mRNAs encoding IFN-γ and IL-10 in vitro, and IFN-γ has been linked to proinflammatory effects [42]. Some studies have suggested that infections caused by pathogens like human cytomegalovirus (CMV) and dengue virus (DENV) lead to an increase in terminal effector CD4+ cells [43,44]. Thus, a higher relative abundance of *Flavonifractor* appears to be harmful to human health. The impact of immune cells on overall health, as well as the increased proinflammatory state, could potentially be modulated through the gut microbiome. However, further research is necessary to identify specific microbial taxa responsible for mediating these effects.

Of interest, we found that all the bacteria found to be associated with the distribution of circulating T cell subsets in the peripheral blood belonged to the *Bacillota* phylum. *Bacillota* bacteria in the gut are known to produce short-chain fatty acids (SCFAs), which have been linked to improvements in immune function [45]. Resistant starch can be broken down into SCFAs by bacteria from the *Bacillota* phylum. The colon predominantly contains three types of SCFAs: acetate, propionate, and butyrate, which serve as a source of energy for epithelial cells and possess properties that reduce inflammation and facilitate immune signaling [45,46]. Our study demonstrated an association between T cell subsets and *Bacillota* bacteria, which suggests the involvement of common biological processes between the regulation of T cells and gut microbiome, although the exact mechanism remains unknown.

### Strengths and Limitations

This is the first study to evaluate the association between circulating T cell subsets in peripheral blood and gut microbial taxa obtained from healthy individuals. By analyzing healthy participants, we had a broad understanding of the connection between T cell subsets and bacterial genera. This approach will facilitate future comparisons across studies. Incorporating bacterial genera at both baseline and after 6 weeks enabled us to detect alterations in the bacterial genera over the course of time. The major limitations of this study are the small sample size and short follow-up time (6 weeks). Since our primary interest was not to evaluate the effect of aspirin on T cell distribution or the gut microbiome but rather to evaluate the association between circulating T cells and the gut microbiome, we analyzed the data by combining both study arms together despite the randomized clinical trial study design to maximize the sample size. Since aspirin intake was not associated with T cell subset distribution at baseline and aspirin intake did not significantly impact the bacterial taxa that were associated with the T cell subsets, combining the two study arms into a single analysis did not introduce any apparent bias in the study results. Furthermore, we adjusted for the study treatment arm (aspirin/placebo) in all regression analyses to minimize any bias in the study results. Another limitation was that 16S rRNA sequencing only provided information on the relative abundance of bacterial taxa and did not provide information on their function or activity. This means that changes in the abundance of specific bacterial taxa over time may not necessarily reflect absolute changes in microbial genera or changes in their metabolic activity or contribution to host health. While 16s sequencing can be informative about the gut microbiome, additional methods, such as metagenomic sequencing, may provide a more comprehensive understanding of longitudinal changes in the gut microbiome, as they can provide more detailed insights into alterations in bacterial genera and their function.

## 4. Material and Methods

### 4.1. Study Design

The study participants were healthy individuals from the greater Minneapolis–St. Paul, Minnesota area, aged 50–75, who were recruited as part of our double-blinded randomized placebo-controlled trial clinical trial—the Effect of Aspirin on Gut Microbiome (ASMIC) study (NCT02761486) [47]. The ASMIC study hypothesized that aspirin intake reduced CRC risk by decreasing the abundance of proinflammatory gut bacteria and increasing anti-inflammatory gut bacteria. The study objective was to compare gut microbiome changes linked to aspirin intake using a double-blinded, placebo-controlled clinical trial design of aspirin intake with 50 healthy volunteers over 6 weeks. Among the 50 study participants, 30 participants received a daily dose of 325 mg of aspirin and 20 received a placebo. The ASMIC study design has been described previously in more detail [47]. We collected blood samples at the baseline visit and stool samples at the baseline and at the 6-week follow-up visit from all participants. We profiled 10 T cell subsets in the blood and gut microbiome from the stool samples.

### 4.2. Collection and Processing of the Stool Samples

Participants collected their stool samples at home using stool collection kits. All stool samples collected at each visit were processed in one batch [47]. DNA was extracted from the stool, and the V4 hypervariable region on the 16S rRNA gene was amplified and sequenced using MiSeq (2 × 300 paired-end; Illumina, San Diego, CA, USA) at the University of Minnesota Genomics Center (UMGC) using protocols published previously [47].

### 4.3. Data Preprocessing

Mothur ver. 1.35.1 was used to process and analyze the sequenced data, and the high-quality sequences were aligned with SILVA database ver. 123.33. UCHIME package (Edgar, 2010) was utilized to remove chimeric sequences. The clustering of OTUs was performed to 97% identify, and the taxonomic classification was completed using version 16 data from the Ribosomal Database Project [47]. Bacterial genera were included for further analyses if they had a non-zero relative abundance among at least 60% of participants at the baseline and at the follow-up visit (high-frequency genera). We included 50 high-frequency bacterial genera in the downstream analyses. Participants with missing microbiome data at baseline (*n* = 1) and at the 6-week visit (*n* = 3) were excluded from the analysis, leaving 47 participants with complete data at two visits.

### 4.4. Collection of Blood Samples and Measurement of T Cell Subsets

Two milliliters (mL) of blood collected in EDTA tubes was incubated for 10 min in 10 milliliters of 1X Red Blood Cell (RBC) lysis buffer (BioLegend, San Diego, CA, USA), centrifuged, and immunophenotyped on the same day using the standardized protocol [35]. The immunophenotyping and differential white blood cell test were conducted by the Advanced Research and Diagnostic Laboratory (ARDL) at the University of Minnesota (Minneapolis, MN, USA). Briefly, the T cells were measured after thawing using standard procedures and stained using the following antibody cocktail fixable viability stain for identifying live/dead cells: FVS570, CD3-APC, CD19-PE-Cy7, IgD-BUV737, HLA-DR-PE-CF594, CD4-APC-Cy7, CD8-BUV395, CD27-FITC, CD127-PE-CF594, CD45RA-BV711 CCR7-BV421, CD95-BV605, and CD28-BV510. We used FlowJo and OpenCyto for the gating and data analysis of all 10 T cell subsets. The major T cell subsets measured were CD4+ T cells (CD3+ CD19− CD4+ CD8−) and CD8+ T cells (CD3+ CD19− CD4− CD8+). The subsets of both CD4+ and CD8+ T cells were defined as follows: activated T cells (HLA− DR+), naïve (CD45RA+ CCR7+ CD28+), central memory (CM: CD45RA− CCR7+ CD28+), effector memory (EM: CD45RA− CCR7− CD28−), and terminal effector (CD45RA+ CCR7− CD28−).

### 4.5. Covariates

Participant’s age, sex, weight (lbs.), and height (inches) were collected by a trained technician at the baseline visit. We estimated the body mass index (BMI) in kg/m^2^ based on the equation weight (lbs.)/[height (inches)]^2^ × 703 if available. A brief baseline questionnaire queried about the intake of meat, yogurt, and fruits and vegetables (servings per week).

### 4.6. Statistical Analysis

The statistical analysis included a cross-sectional analysis, which examined associations between T cell subsets and bacterial genera at baseline, and a longitudinal analysis of the 6-week changes in the microbiome, which examined associations between the baseline percentages of T cell subsets and the fold changes in the relative abundances of the bacterial genera between 6 weeks and at baseline. All 10 T cell subsets had a normal distribution. We evaluated the association between T cell subsets and aspirin intake at baseline. To reduce the impact of outliers due to the skewed data and to approximate normal distribution, log transformation and standardization were applied to all bacterial genera.

### 4.7. Sparse Canonical Correlation Analysis (sCCA)

We used sparse canonical correlation analysis (sCCA) to identify linear projections of group-level correlations between T cell subsets and the gut microbiome. sCCA has been used in several studies for multi-omics data integration and analysis [48]. Sparse CCA is an adapted version of CCA for feature selection after applying L1 or lasso penalty in CCA in the high-dimensional feature space. The sCCA analysis was conducted using the Penalized Multivariate Analysis (PMA) package in R [49], which resulted in sparsity through penalized matrix decomposition with Lasso penalty (L1). The L1 parameter was tuned using a permutation approach in the CCA.permute function in R, where a grid search ranging from 0.1 to 0.7 was used to find the optimal L1 value that maximized the correlation between the microbiome and T cell subsets at a significance level of 0.05. We applied the L1 value of 0.367 on the T cell subsets and 0.433 on the bacterial taxa for the cross-sectional analysis and 0.5 on the T cell subset and 0.7 on the bacterial taxa for the longitudinal analysis. The objective function of sCCA is shown below (Equation (1)) [49]. We performed a permutation test (number of permutations = 1000) at the 0.05 significance level.

(1) $${{maxmize}_{u,v}u^{T}X^{T}Yv\;subject\;to\left|\left|u\right|\right|}_{2}^{2}\leq 1,{\left|\left|v\right|\right|}_{2}^{2}\leq 1,P_{1}\left(u\right)\leq c_{1,}P_{2}\left(v\right)\leq c_{2,}$$

$Note:P_{1}\;and\;P_{2}\;are\;convex\;functions.$

### 4.8. Linear Regression Analysis

We selected significant pairs of canonical variates to select pairs of bacterial genera and T cell subsets for further association analysis. The multivariable linear regression models were adjusted for age, sex, BMI, and fruit and vegetable servings per week in the cross-sectional analysis and for the treatment arm (aspirin/placebo). In addition, we adjusted for the baseline bacterial genera measurements in the longitudinal analysis. We considered a *p*-value of ≤0.01 for an association between a T cell subset and bacterial genera to be statistically significant in the regression analysis.

## 5. Conclusions

In conclusion, our study provided preliminary evidence that multiple T cell subsets are associated with relative change in bacterial genera over 6 weeks of follow-up time. Future experimental studies with longer longitudinal follow-up are needed to focus on the role of circulating T cells in determining the gut microbiome composition and thereby maintaining gut homeostasis. The bacterial genera in the *Bacillota* phylum were predominantly associated with various T cell subsets in both the cross-sectional and longitudinal change analyses after adjusting for covariates. This might suggest that the *Bacillota* being major producers of short-chain fatty acids (SCFAs) could potentially play a crucial role in the homeostasis of the gut microbiome. These study findings are particularly important in settings where a mechanistic understanding of the role of adaptive immune T cell subsets is critical, such as in the prevention and treatment of immune-related diseases, including cancer, inflammatory bowel disease, allergies, and autoimmune disorders.

## Figures and Tables

**Table 1:** Association between the T cell subsets and bacterial genera identified from significant canonical variates in the cross-sectional analysis.

Immune Cell	Estimate	Standard Error	*p*-Value	Bacterial Genera (Phylum)
Naïve CD4+	0.03	0.01	<0.01	*Intestinimonas (Bacillota)*
Activated CD8+	−0.11	0.05	0.03	*Cellulosibacter (Bacillota)*

**Table 2:** Association between the T cell subsets and microbiome identified from significant canonical variates in the longitudinal analysis.

Immune Cell	Estimate	Standard Error	*p*-Value	Bacterial Genera (Phylum)
Activated CD4+	−0.07	0.03	0.03	*Anaerovorax (Bacillota)*
Terminal Effector CD4+	0.05	0.02	0.01	*Flavonifractor (Bacillota)*
Naïve CD4+	−0.03	0.03	0.02	*Clostridium_XlVb (Bacillota)*

**Table 3:** Common significant and nominal combinations of bacterial genera and immune cells between two analyses.

Microbiome	Immune Cells and Time Point		Estimate (*p*-Value)
*Intestinimonas*	Naïve CD4+	Baseline	0.03 (<0.01)
*Clostridium_XlVb*		6-weeks change	−0.03 (0.02)

## Data Availability

The data that support the findings of this study are available upon request from the corresponding author (B.T.). The data are not publicly available due to the regulations on clinical trial data.

## Supplementary Materials

The following supporting information can be downloaded at: https://www.mdpi.com/article/10.3390/ijms25136831/s1.
