# Supplementary material for: Association between Circulating T Cells and the Gut Microbiome in Healthy Individuals: Findings from a Pilot Study
Source: Int J Mol Sci. 2024 Jun 21;25(13):6831. doi: 10.3390/ijms25136831 (PMC11241708; doi:10.3390/ijms25136831)
Supplement: Supplementary file 1 [file ijms-25-06831-s001.zip › ijms-3036914-supplementary.pdf]

Supplementary Table S1. Results of cross-sectional analysis using sparse Canonical Correlation Analysis (sCCA), *-*p* value.

**Baseline (X L1 penalty 0.367, Z L1 penalty : 0.433)**		
**Pearson (p)**	Baseline T cell (X)	Baseline bacterial genera with log transformation (Z)
**K1: 0.69 (0.007*)**	Activated CD4+ (-0.984)<br>Effector memory CD4+ (-0.176)	*Phascolarctobacterium, Parabacteroides, Roseburia, Sutterella, Blautia, Oscillibacter, Ruminococcus, Collinsella, Lachnospira, Ruminococcus2, Fusicatenibacter, Anaerostipes, Dorea, Anaerotruncus, Coprococcus, Acetitomaculum, Butyrivibrio, Cellulosibacter, Butyricicoccus* (N = 19)<br>0.004, 0.006, 0.584, 0.080, -0.090, 0.169, 0.176, 0.065, 0.029, 0.167, 0.133, -0.070, -0.031, 0.284, 0.562, 0.086, 0.110, 0.094, -0.322
**K2: 0.55 (0.074)**	Central memory CD8+ (-0.176)<br>Terminal effector CD8+ (0.984)	*Bacteroides, Alistipes, Akkermansia, Roseburia, Oscillibacter, Parasutterella, Anaerostipes, Dorea, Anaerotruncus, Bilophila, Acetitomaculum, Clostridium_sensu_stricto, Sporobacter, Haemophilus, Intestinimonas*(N = 15)<br>0.335, -0.433, -0.463, 0.252, -0.090, 0.012, -0.318, -0.014, -0.387, -0.055, -0.031, -0.107, -0.029, 0.234, -0.301

**K3: 0.64 (0.003*)**	Central memory CD4+ (0.984)<br>Naïve CD4+ (-0.176)	*Faecalibacterium, Parabacteroides, Oscillibacter, Parasutterella, Collinsella, Bifidobacterium, Anaerostipes, Dorea, Streptococcus, Anaerosporobacter, Butyrivibrio, Cellulosibacter, Lachnobacterium, Intestinimonas, Asaccharobacter* (N = 15)<br>-0.440, 0.037, -0.092, 0.089, 0.278, 0.057, 0.292, 0.054, 0.234, -0.182, 0.436, -0.199, -0.536, -0.122, -0.013
**K4: 0.51 (0.282)**	Effector memory CD8+ (0.176)<br>Naïve CD8+ (-0.984)	*Phascolarctobacterium, Faecalibacterium, Akkermansia, Lachnospira, Anaerostipes, Flavonifractor, Anaerotruncus, Bilophila, Streptococcus, Clostridium_sensu_stricto, Veillonella, Romboutsia, Anaerosporobacter, Butyrivibrio, Lachnobacterium, Intestinimonas, Anaerovorax* (N = 17)<br>-0.198, -0.152, -0.204, -0.071, 0.063, -0.083, -0.140, -0.423, 0.056, -0.178, -0.195, -0.108, -0.071, -0.116, -0.105, -0.167, -0.735
**K5: 0.60 (0.045*)**	Activated CD8+ (-0.984)<br>Effector memory CD8+ (-0.176)	*Phascolarctobacterium, Parasutterella, Collinsella, Lachnospira, Anaerostipes, Coprococcus, Clostridium_XVIII, Bilophila, Intestinibacter, Haemophilus, Butyrivibrio, Cellulosibacter, Christensenella, Anaerovorax, Butyricicoccus* (N =15)

		0.321, 0.010, 0.163, 0.223, -0.199, 0.295, 0.248,<br>0.107, -0.564, 0.082, 0.141, 0.105, -0.025, 0.068, -0.510
**K6:**<br>**0.54**<br>**(0.289)**	Effector memory CD8+ (-0.984)<br>Naïve CD4+ (-0.176)	*Phascolarctobacterium, Oscillibacter, Parasutterella, Collinsella, Bifidobacterium, Ruminococcus2, Fusicatenibacter, Anaerostipes, Dorea, Anaerotruncus, Coprococcus, Clostridium_XVIII, Bilophila, Clostridium_XlVb, Veillonella, Intestinibacter, Haemophilus, Christensenella, Asaccharobacter* (N = 19)<br>0.037, -0.237, 0.253, 0.170, 0.004, -0.073, 0.090,<br>-0.025, 0.147, -0.016, 0.038, 0.155, 0.075, -0.403, 0.283,<br>0.044, 0.638, -0.012, -0.363
**K7:**<br>**0.54**<br>**(0.132)**	Terminal effector CD4+ (-0.984)<br>Naïve CD4+ (0.176)	*Faecalibacterium, Akkermansia, Clostridium_XlVa, Roseburia, Blautia, Oscillibacter, Clostridium_IV, Anaerotruncus, Acetitomaculum, Subdoligranulum, Cellulosibacter, Intestinimonas, Butyricicoccus, Asaccharobacter* (N =14)<br>-0.010, 0.285, -0.226, -0.106, 0.117, 0.014, 0.296, 0.478,<br>0.026, 0.398, 0.226, 0.175, -0.490, 0.214

**K8:**<br>**0.56**<br>**(0.067)**	Effector memory CD4+ (0.984)<br>Naïve CD4+ (-0.176)	*Clostridium_XlVa, Sutterella, Oscillibacter, Ruminococcus, Bifidobacterium, Ruminococcus2, Anaerotruncus, Acetitomaculum, Sporobacter, Anaerosporobacter, Haemophilus, Cellulosibacter, Lachnobacterium, Intestinimonas, Butyricicoccus, Pseudobutyrivibrio* (N=16)<br>0.060, -0.340, -0.231, -0.061, -0.012, -0.030, -0.531, -0.151, -0.191, -0.103 0.025, -0.489, -0.315, -0.207, 0.298, -0.018
**K9:**<br>**0.47**<br>**(0.28)**	Activated CD4+ (-0.984)<br>Effector memory CD4+ (-0.176)	*Phascolarctobacterium, Parabacteroides, Roseburia, Collinsella, Bifidobacterium, Lachnospira, Gemmiger, Anaerostipes, Flavonifractor, Dorea, Bilophila, Sporobacter, Pseudobutyrivibrio* (N =13)<br>0.489, 0.479, -0.044, 0.071, 0.288, 0.321, 0.262, -0.035, 0.181, -0.306, 0.321, 0.175, 0.088
**K10:**<br>**0.55**<br>**(0.086)**	Activated CD4+ (0.176)<br>Naïve CD4+ (-0.984)	*Faecalibacterium, Oscillibacter, Ruminococcus, Gemmiger, Anaerostipes, Anaerotruncus, Streptococcus, Sporobacter, Anaerosporobacter, Haemophilus, Butyrivibrio, Cellulosibacter, Lachnobacterium, Intestinimonas, Butyricicoccus* (N= 15)

		-0.039, -0.276, -0.242, -0.023, 0.218, -0.002, 0.117,<br>-0.146, -0.238, 0.161, -0.268, -0.251, -0.652, -0.367,<br>0.062

Supplementary Table S2. Results of longitudinal analysis using sparse Canonical Correlation Analysis (sCCA) **Longitudinal (x L1 bound 0.7, z: L1 bound : 0.5), *-*p* value.**

**Pearson**<br>**(p)**	T cell	Microbiome
**K1: 0.732**<br>**(0.006*)**	Central memory CD8+(-0.677)<br>Terminal Effector CD8+ (0.407)<br>Activated CD4+ (-0.347)<br>Central memory CD4+ (-0.397)<br>Terminal Effector CD4+ (0.303)<br>Naïve CD4+ (0.083)	*Alistipes 0.221 Parabacteroides 0.242 Clostridium_XlVa 0.166 Collinsella 0.001 Lachnospira -0.12 Fusicatenibacter 0.385 Anaerostipes 0.256 Flavonifractor -0.316 Dorea 0.422 Clostridium_XVIII 0.441 Acetitomaculum 0.214 Streptococcus 0.147 Clostridium_XlVb 0.265 Veillonella 0.023 Anaerosporobacter 0.067 Butyrivibrio -0.065 Anaerovorax -0.111 Asaccharobacter 0.074 (N = 18)*

**K2: 0.595 (0.274)**	Terminal Effector CD8+ (0.551)<br>Naïve CD8+ (-0.464)<br>Central memory CD4+ (0.306)<br>Terminal Effector CD4+ (0.179)<br>Effector Memory CD4+ (0.132)<br>Naïve CD4+ (-0.581)	*Phascolarctobacterium -0.014 Faecalibacterium 0.226 Roseburia 0.059 Sutterella 0.099 Blautia 0.49 Oscillibacter -0.019 Parasutterella -0.274 Ruminococcus 0.005 Odoribacter -0.299 Gemmiger 0.03 Anaerostipes -0.074 Anaerotruncus 0.151 Acetitomaculum 0.076 Streptococcus 0.056 Clostridium_sensu_stricto 0.462 Clostridium_XlVb -0.063 Romboutsia 0.115 Intestinibacter 0.002 Subdoligranulum -0.096 Christensenella 0.017 Lachnobacterium 0.058 unclassified -0.388 Intestinimonas -0.122 Pseudobutyrivibrio -0.283 Hungatella -0.057 (N = 25)*
**K3: 0.631 (0.218)**	Activated CD8+ (-0.741)<br>Central memory CD8+ (-0.096)<br>Terminal Effector CD8+ (0.312)<br>Effector Memory CD8+ (-0.484)<br>Naïve CD8+ (0.12)<br>Activated CD4+ (-0.289)<br>Central memory CD4+ (-0.036)<br>Terminal Effector CD4+ (-0.011)<br>Effector Memory CD4+ (0.104)<br>Naïve CD4+ (0.002)	*Alistipes 0.314 Phascolarctobacterium -0.457 Clostridium_XlVa 0.072 Roseburia 0.101 Oscillibacter 0.209 Clostridium_IV 0 Parasutterella -0.165 Ruminococcus 0.334 Collinsella -0.06 Lachnospira 0.07 Ruminococcus2 0.202 Dorea 0.085 Clostridium_XVIII -0.09 Clostridium_XlVb 0.278 Intestinibacter 0.181 Haemophilus -0.02 Christensenella 0.432 Intestinimonas 0.351 Anaerovorax -0.002 Pseudobutyrivibrio 0.099 Hungatella -0.015 (N = 21)*

**K4: 0.577 (0.503)**	Activated CD8+ (0.279)<br>Central memory CD8+ (-0.047)<br>Terminal Effector CD8+ (0.047)<br>Effector Memory CD8+ (0.149)<br>Naïve CD8+ (-0.168)<br>Activated CD4+ (-0.701)<br>Central memory CD4+ (0.135)<br>Effector Memory CD4+ (-0.59)<br>Naïve CD4+ (0.098)	*Bacteroides -0.053 Alistipes 0.09 Akkermansia -0.022 Parabacteroides -0.173 Clostridium_XlVa 0.364 Roseburia -0.018 Sutterella -0.019 Blautia -0.126 Parasutterella 0.092 Odoribacter 0.039 Fusicatenibacter -0.022 Flavonifractor -0.209 Acetitomaculum -0.134 Streptococcus 0.209 Clostridium_sensu_stricto 0.195 Veillonella 0.536 Intestinibacter 0.244 Haemophilus 0.49 Cellulosibacter -0.113 Lachnobacterium -0.023 unclassified 0.204 Anaerovorax 0.1 Hungatella 0.061 (N = 23)*
**K5:0.506 (0.968)**	Activated CD8+ (0.437)<br>Central memory CD8+ (-0.462)<br>Terminal Effector CD8+ (0.163)<br>Activated CD4+ (0.153)<br>Central memory CD4+ (-0.26)<br>Terminal Effector CD4+ (0.69)<br>Effector Memory CD4+ (0.022)<br>Naïve CD4+ (-0.026)	*Bacteroides -0.027 Alistipes -0.015 Phascolarctobacterium -0.278 Akkermansia 0.08 Parabacteroides 0.066 Clostridium_XlVa -0.265 Roseburia 0.012 Blautia 0.034 Oscillibacter -0.027 Collinsella 0.48 Lachnospira -0.554 Dorea -0.198 Coprococcus 0.151 Bilophila 0.014 Streptococcus 0.064 Sporobacter -0.345 Veillonella 0.132 Anaerosporobacter 0.082 Haemophilus 0.129 Subdoligranulum -0.091 Butyrivibrio -0.17 Cellulosibacter 0.066 unclassified -0.154 Butyricicoccus -0.103 (N = 24)*

**K6:0.566 (0.561)**	Activated CD8+ (-0.219)<br>Central memory CD8+ (-0.282)<br>Terminal Effector CD8+ (0.208)<br>Naïve CD8+ (-0.141)<br>Activated CD4+ (-0.004)<br>Central memory CD4+ (-0.737)<br>Effector Memory CD4+ (0.12)<br>Naïve CD4+ (0.501)	*Phascolarctobacterium 0.101 Akkermansia 0.46 Clostridium_XlVa 0.4 Roseburia 0.23 Blautia 0.042 Clostridium_IV 0.106 Bifidobacterium 0.334 Gemmiger -0.063 Anaerostipes 0.273 Anaerotruncus -0.069 Bilophila 0.125 Acetitomaculum 0.063 Romboutsia -0.081 Subdoligranulum 0.001 Butyrivibrio -0.124 Lachnobacterium -0.187 unclassified -0.369 Pseudobutyrivibrio 0.165 Asaccharobacter 0.344 (N = 19)*
**K7:0.505 (0.991)**	Activated CD8+ (0.465)<br>Central memory CD8+ (0.384)<br>Effector Memory CD8+ (-0.727)<br>Naïve CD8+ (0.248)<br>Central memory CD4+ (-0.097)<br>Terminal Effector CD4+ (0.037)<br>Effector Memory CD4+ (-0.169) Naïve CD4+ (0.087)	*Phascolarctobacterium -0.124 Faecalibacterium -0.071 Akkermansia -0.181 Parabacteroides -0.038 Roseburia -0.007 Sutterella -0.114 Collinsella -0.512 Bifidobacterium -0.136 Fusicatenibacter 0.057 Flavonifractor -0.021 Dorea 0.077 Anaerotruncus -0.166 Clostridium_XVIII -0.169 Clostridium_XlVb 0.021 Sporobacter -0.213 Intestinibacter -0.431 Subdoligranulum -0.251 Cellulosibacter -0.089 Christensenella -0.048 Lachnobacterium -0.333 Butyricicoccus -0.407 Hungatella 0.068 (N = 22)*

**K8:0.332**<br>**(> 0.99)**	Activated CD8+ (0.637)<br>Central memory CD8+ (0.285)<br>Effector Memory CD8+ (0.308)<br>Naïve CD8+ (-0.523)<br>Activated CD4+ (0.368)<br>Terminal Effector CD4+ (-0.092)	*Faecalibacterium -0.241 Parabacteroides 0.218 Blautia -0.085 Oscillibacter -0.243 Clostridium_IV -0.329 Parasutterella 0.041 Odoribacter -0.211 Anaerostipes 0.289 Flavonifractor -0.077 Coprococcus -0.147 Clostridium_sensu_stricto -0.435 Romboutsia 0.356 Intestinibacter -0.014 Anaerosporobacter -0.135 Cellulosibacter -0.022 Christensenella 0.202 Anaerovorax 0.429 Pseudobutyrivibrio -0.011 Hungatella 0.05 (N = 19)*
**K9:0.263**<br>**(> 0.99)**	Activated CD8+ (-0.045)<br>Central memory CD8+ (-0.417)<br>Terminal Effector CD8+ (0.753)<br>Effector Memory CD8+ (-0.289) Naïve CD8+ (-0.229)<br>Activated CD4+ (-0.01)<br>Effector Memory CD4+ (0.307) Naïve CD4+ (-0.164)	*Faecalibacterium 0.005 Parabacteroides 0.143 Clostridium_XlVa 0.005 Roseburia 0.398 Oscillibacter -0.428 Clostridium_IV -0.141 Lachnospira 0.032 Ruminococcus2 0.05 Anaerostipes -0.109 Coprococcus 0.382 Clostridium_XVIII -0.49 Acetitomaculum 0.191 Clostridium_XlVb -0.155 Sporobacter -0.174 Veillonella 0.143 Intestinibacter 0.002 Anaerosporobacter 0.091 Intestinimonas -0.213 Anaerovorax -0.167 Butyricicoccus 0.091 Hungatella -0.126 (N = 21)*

**K10: 0.543 (0.725)**	Activated CD8+ (-0.569)<br>Central memory CD8+ (0.19)<br>Naïve CD8+ (-0.224)<br>Activated CD4+ (-0.364)<br>Central memory CD4+ (-0.231)<br>Terminal Effector CD4+ (0.636)	*Bacteroides -0.057 Phascolarctobacterium 0.204 Parabacteroides 0.034 Sutterella 0.017 Clostridium_IV 0.172 Parasutterella -0.081 Odoribacter 0.271 Collinsella -0.046 Ruminococcus2 0.05 Gemmiger 0.069 Dorea 0.11 Acetitomaculum 0.383 Intestinibacter 0.055 Anaerosporobacter 0.166 Haemophilus -0.058 Butyrivibrio -0.508 Cellulosibacter 0.438 Lachnobacterium -0.223 Butyricicoccus -0.173 Asaccharobacter 0.102 Hungatella -0.319 (N = 21)*
